# A slowly progressive form of limb-girdle muscular dystrophy type 2C associated with founder mutation in the *SGCG* gene in Puerto Rican Hispanics

**DOI:** 10.1002/mgg3.125

**Published:** 2015-01-08

**Authors:** Samiah A Al-Zaidy, Vinod Malik, Kelley Kneile, Xiomara Q Rosales, Ana Maria Gomez, Sarah Lewis, Sayaka Hashimoto, Julie Gastier-Foster, Peter Kang, Basil Darras, Louis Kunkel, Jose Carlo, Zarife Sahenk, Steven A Moore, Robert Pyatt, Jerry R Mendell

**Affiliations:** 1 Center for Gene Therapy and Paul D. Wellstone Muscular Dystrophy Research Center, Nationwide Children's HospitalColumbus, Ohio; 2 Department of Pediatrics and Neurology, The Ohio State UniversityColumbus, Ohio; 3 Department of Pathology, Ohio State University and Nationwide Children's HospitalColumbus, Ohio; 4 Department of Pathology and Laboratory Medicine, Nationwide Children's HospitalColumbus, Ohio; 5 Department of Neurology, Boston Children's Hospital and Harvard Medical SchoolBoston, Massachusetts; 6 Division of Genetics and Genomics, The Manton Center for Orphan Disease Research, Boston Children's HospitalBoston, Massachusetts; 7 Department of Neurology, School of Medicine, University of Puerto RicoSan Juan, Puerto Rico; 8 Department of Pathology, The University of Iowa Carver College of MedicineIowa City, Iowa

**Keywords:** Founder mutation, gamma-sarcoglycan, LGMD2C, Puerto Rico, *SGCG*

## Abstract

Limb-girdle muscular dystrophy type 2C (LGMD2C) is considered one of the severe forms of childhood-onset muscular dystrophy. The geographical distribution of founder mutations in the *SGCG* gene has a prominent effect on the prevalence of LGMD2C in certain populations. The aim of this study was to confirm the hypothesis that the c.787G>A (p.E263K) mutation in the *SGCG* gene is a founder mutation among Puerto Rican Hispanics and to characterize the associated clinical and immunohistochemical phenotype. Genotyping of six polymorphic microsatellite markers internal to (D13S232) and flanking (D13S175, D13S292, D13S787, D13S1243, D13S283) the *SGCG* gene was performed on four unrelated Puerto Rican patients with LGMD2C. Preserved ambulation to the second decade of life was observed in at least two subjects. Immunostaining of skeletal muscle demonstrated absence of *γ*-sarcoglycan in all affected subjects. Two markers, D13S232 and D13S292, were highly informative and confirmed that all four families share the haplotype of the mutant allele. Our findings confirm that the E263K missense mutation in the *SGCG* gene is a founder mutation in Puerto Rican Hispanics. A slowly progressive disease course with prolonged preservation of ambulation can be seen in association with this mutation, providing evidence for phenotypic variability.

## Introduction

The limb-girdle muscular dystrophies (LGMDs) are a clinically and genetically heterogeneous group of disorders. The sarcoglycanopathies form a subgroup of autosomal recessive LGMDs (LGMD2C-F) that result from mutations in the genes encoding *γ*-, *α*-, *β*-, and *δ*-sarcoglycan, respectively. These transmembrane glycoproteins form the sarcoglycan complex, which plays a fundamental role in stabilizing the sarcolemmal membrane through its interaction with the dystrophin-associated glycoprotein (DAG) complex. Deficiency of any of the four subunits results in perturbation of the sarcoglycan complex and manifests clinically as a progressive muscular dystrophy. Although the *α*- and *β*-sarcoglycan LGMD subtypes have a greater overall prevalence (Duggan et al. 1997; Fanin et al. 1997; Moreira et al. 2003; Zatz et al. 2003), the geographical distribution of founder mutations in the gamma-sarcoglycan gene, *SGCG* has a prominent effect on the prevalence of LGMD2C in certain populations such as the North African c.525delT p.(F175Lfs*20) and European Gypsy c.848G>A p.(C283Y) LGMD2C patients (Ben Hamida et al. 1983; Piccolo et al. 1996; Lasa et al. 1998).

Limb-girdle muscular dystrophy type 2C (LGMD2C) is an autosomal recessive disease caused by mutations in the *SGCG* gene (OMIM#253700) and subsequent deficiency of the gamma-sarcoglycan protein (Noguchi et al. 1995; McNally et al. 1996a). Due to its clinical resemblance to the X-linked Duchenne muscular dystrophy (DMD) (Ben Hamida et al. 1983; Kefi et al. 2003), LGMD2C has been previously described as autosomal recessive Duchenne-like muscular dystrophy and severe childhood autosomal recessive muscular dystrophy (SCARMD) (Azibi et al. 1993). It typically presents in childhood with progressive muscle weakness, calf hypertrophy, elevated serum creatine kinase (CK), and loss of ambulation early in the second decade of life (Kirschner and Lochmuller 2011). However, variability in the severity of weakness has been described across LGMD2C patients with the same mutation (McNally et al. 1996b), especially in the North African c.525delT founder mutation (Kefi et al. 2003).

In this report, we describe two unrelated LGMD2C patients of Hispanic descent from the island of Puerto Rico that share a c.787G>A (p.E263K) mutation within exon 8 of the *SGCG* gene (Duncan et al. 2006; Dicapua and Patwa 2014) and a slowly progressive form of LGMD2C with prolonged preservation of ambulation. We also provide evidence that this mutation is a founder mutation among Puerto Rican Hispanics.

## Patients and Methods

### Patients

Two unrelated families from Puerto Rico with a confirmed c.787G>A (E263K) mutation in the *SGCG* gene were included in this study. Index patients (Patients 1 and 2) underwent a complete clinical evaluation that included a neuromuscular examination, cardiac and pulmonary evaluations, serum CK levels and muscle biopsies. In addition to our two patients, DNA from the two previously reported families (Patients 3 and 4 in this study) was obtained from Boston Children's Hospital (kindly provided by Dr. Peter Kang). Clinical, histologic and mutation data on Patients 3 and 4 were previously described elsewhere (Duncan et al. 2006). This study was approved by local ethics committees and all subjects consented to participate.

### Muscle biopsy

Skeletal muscle biopsies were obtained from the quadriceps muscles on Patients 1 and 2. Snap frozen sections were evaluated with standard histochemical stains that included hematoxylin and eosin (H&E), modified Gomori trichrome, SDH, NADH, and adenosine triphosphate at pH 4.3, 4.6, and 9.4. Because of the multicenter recruitment of patients and samples, immunohistochemical studies varied slightly. Immunofluorescence stains were done using the following antibodies: carboxyl terminus of dystrophin (Leica: NCL-DYS2 for Patient 1 and Abcam: ab15277 for Patient 2), and the sarcoglycans *α*- (Developmental Studies Hybridoma Bank, The University of Iowa), *β*-, *γ*-, and *δ*-sarcoglycan (Leica Biosystems, Newcastle Upon Tyne, UK).

### DNA preparation and sequencing

After informed consent, blood was drawn on all affected individuals and available family members. Genomic DNA was isolated using the Gentra Puregene Blood Kit (Qiagen Inc, Germantown, MD). Polymerase chain reaction (PCR) amplification and sequencing of all eight exons of the *SGCG* gene was performed for index cases. Targeted mutation analysis was subsequently completed for family members.

### Linkage analysis

Isolated DNA was available on Patient 1 and her parents and two half-siblings and on Patient 2 and his mother. Genotyping of six polymorphic markers internal to (D13S232) and flanking (D13S175, D13S292, D13S787, D13S1243, D13S283) the *SGCG* gene was performed (Table1). In all cases, PCR products were separated by capillary electrophoresis using an AB 3130xl Genetic Analyzer and data were evaluated using GeneMapper v4.0 (Applied Biosystems, California, USA).

**Table 1:** The primers used for amplification of the microsatellite markers

STS marker	Forward primer (5′-3′)	Reverse primer (5′-3′)
D13S232	TGCTCACTGCTCTTGTGATT	GGCACAGAAATAAATGTTGATG
D13S292	TAATGGCGGACCATGC	TTTGACACTTTCCAAGTTGC
D13S175	TATTGGATACTTGAATCTGCTG	TGCATCACCTCACATAGGTTA
D13S787	ATCAGGATTCCAGGAGGAAA	ACCTGGGAGTCGGAGCTC
D13S1243	TGCTGACAGGCTACAGAACTTT	CTCTTGTGCAGGTATAGGGG
D13S283	TCTCATATTCAATATTCTTACTGCA	GCCATTCCAAGCGTGT

## Results

### Clinical phenotype

**Figure 1:**
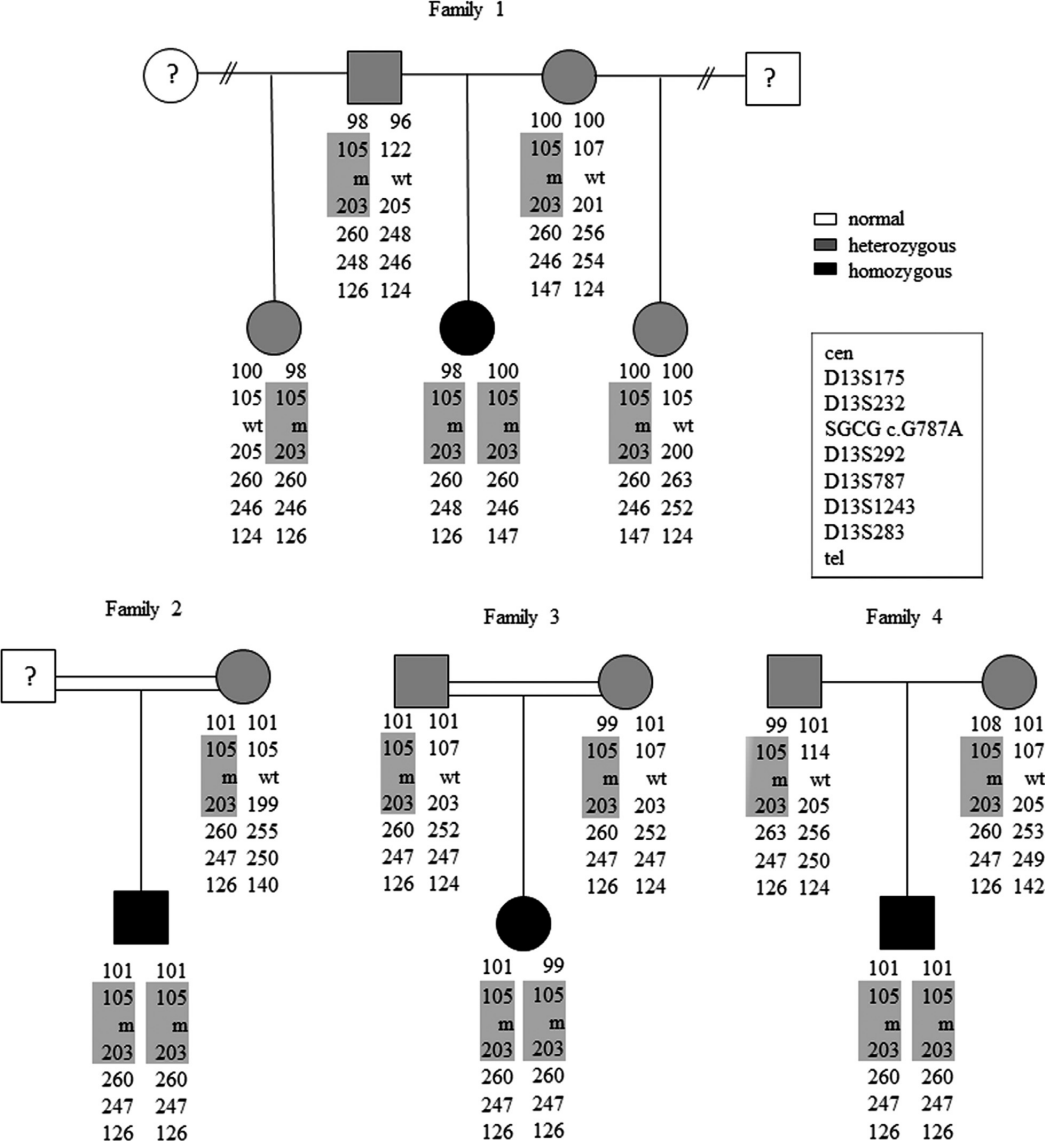
Family structure, genotyping, and haplotype analysis. Two-generation pedigrees for families 1, 2, 3, and 4 are shown. Probands and family members were screened for the E263K mutation. Genotyping for six polymorphic microsatellite markers on chromosome 13 internal to (D13S232) and flanking (D13S175, D13S292, D13S787, D13S1243, D13S283) the *SGCG* gene revealed a common disease-bearing haplotype (shaded in gray).

Our first subject, Patient 1, is an 11-year-old girl who developed proximal muscle weakness at the age of 6 years. This manifested clinically as falling episodes and inability to participate in sports. Motor milestones were normal and there was no cognitive impairment. She has two healthy half-sisters and her parents are nonconsanguineous and of Hispanic background from Puerto Rico (Fig.1). The neuromuscular examination revealed proximal weakness that was more apparent in pelvic girdle muscles. She walked with a lordotic posture but required no assistance in ambulation. She had bilateral calf hypertrophy and mild scapular winging but no joint contractures. Muscle stretch reflexes were easily elicited in all four extremities. Her CK level was extremely high at 18,269 U/L. Results of cardiac tests including electrocardiogram (EKG), echocardiogram, and pulmonary function tests were all normal.

Patient 2, an 11 year-old boy at the time of his initial evaluation, presented with a 2-year history of frequent falling and inability to run. He was an only child born to consanguineous healthy parents from Puerto Rico. Muscle weakness was predominantly proximal with pelvic girdle muscles affected more than upper extremities. Additional findings included bilateral calf hypertrophy, scapular winging, lordosis, and a waddling gait. He had macroglossia but no facial weakness. His CK levels were elevated, ranging between 4500 and 13,560 U/L. Cardiac evaluation by EKG and echocardiogram revealed intact function. By 18 years of age, as a college student, he remained ambulatory, was able to climb stairs with assistance and required no ventilator assistance.

### Muscle histology

**Figure 2:**
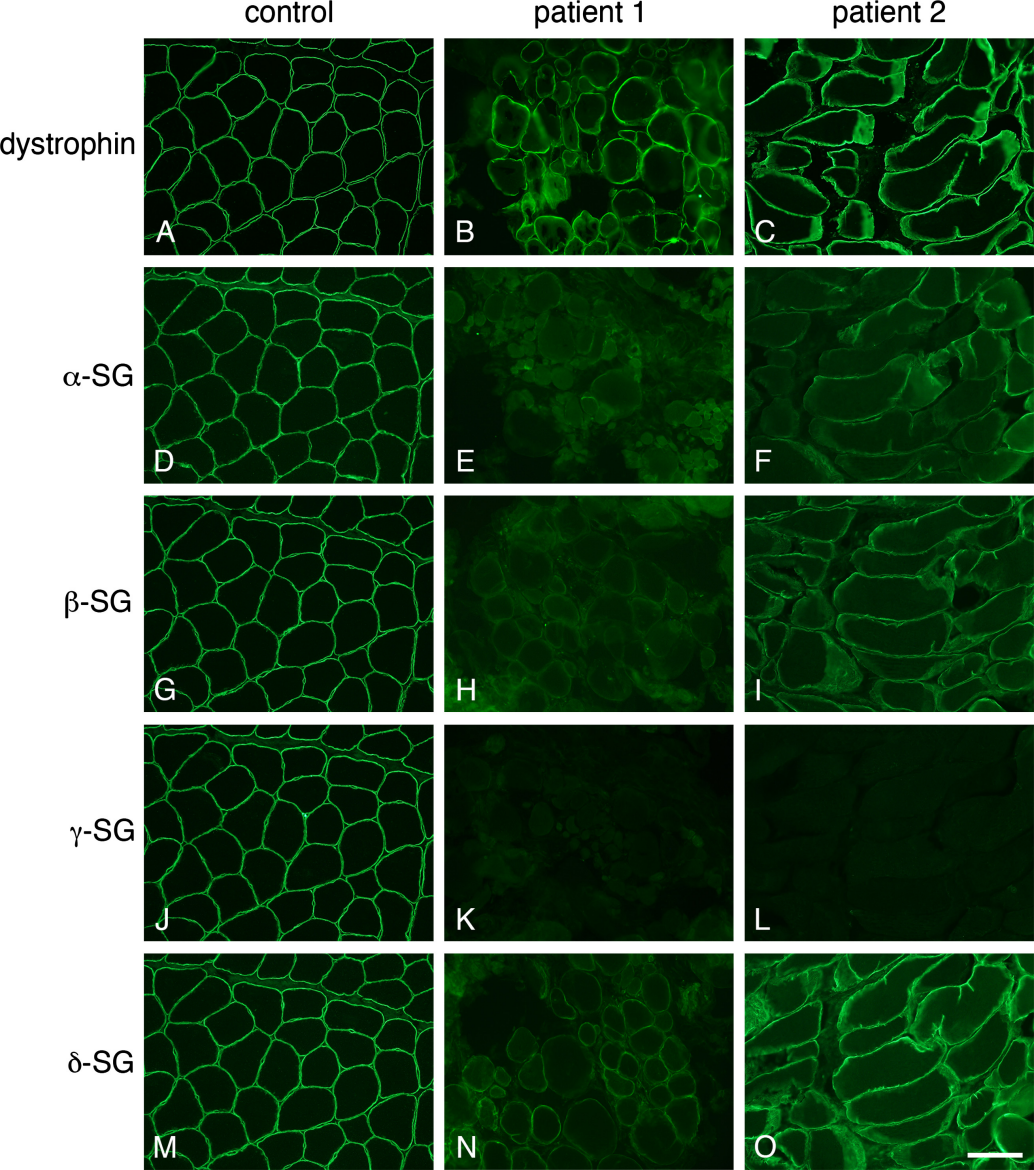
Immunostaining of quadriceps muscle samples for Patients 1 and 2. Complete absence of staining (K and M) for *γ*-sarcoglycan antibody is demonstrated in both patients. Patient 1′s muscle tissue (E, H, and N) shows partial reduction in staining of *α*-, *β*-, and *δ*-sarcoglycan, while tissue on Patient 2 (F, I, and O) demonstrates loss of expression of *α*- and *β*-sarcoglycan and reduction in *δ*-sarcoglycan.

Muscle biopsies for patients 1 and 2 both showed a dystrophic pattern on H&E including marked variability in myofiber size and shape, increased numbers of fibers with internal nuclei as well as fiber splitting. Clusters of necrotic fibers and increased endomysial connective tissue were evident in both samples. Immunostaining of the myofiber membranes was completely absent to the *γ*-sarcoglycan antibody for both patients. In Patient 1, immunostaining for *α*- and *β*-sarcoglycan was absent and reduced for *δ*-sarcoglycan. Muscle tissue on Patient 2 showed patchy weak immunoreactivity to *α*- and *β*-sarcoglycan antibodies and preserved staining to *δ*-sarcoglycan. Antibodies to the carboxyl terminus of dystrophin demonstrated normal membrane staining for both patients (Fig.2).

### Mutation analysis

Targeted amplification and sequence analysis of exon 8 of the *SGCG* gene revealed homozygosity for a previously reported pathogenic missense mutation in all four index cases. The guanine to adenine change at base 787 (c. 787G>A) results in a glutamine to lysine substitution at amino acid 263 (E263K). Unaffected family members were found to be heterozygous carriers of this mutation through targeted genomic sequencing of DNA (Fig1).

### Haplotype analysis

Genotype analysis of six polymorphic markers, D13S175, D13S232, D13S292, D13S787, D13S1243, and D13S283 on all four family members confirmed linkage to the *SGCG* locus on chromosome 13. These markers showed that all four families shared a common allele haplotype including the E263K missense mutation (Fig1).

## Discussion

The LGMD2C is generally considered one of the severe forms of childhood onset autosomal recessive LGMD. The earliest reports of SCARMD in a North African population described a uniform phenotype of proximal muscle weakness that is rapidly progressive with loss of ambulation by the age of 12–15 years (Ben Hamida et al. 1983). Nonetheless, milder forms of LGMD2C do exist (van Kooi et al. 1998), and the same *SGCG* mutation can produce heterogeneous phenotypes that vary in severity (McNally et al. 1996b). In contrast, reports of a missense mutation (C283Y) were associated with a consistently severe phenotype of LGMD2C in the Western European Gypsy population (Piccolo et al. 1996; Lasa et al. 1998; Merlini et al. 2000). In Duncan et al. (2006) reported on two unrelated LGMD2C patients of Puerto Rican descent who harbored the same missense c.787G>A (p.E263K) mutation. One of the two subjects had lost ambulation at the age of 10 years indicating a more severe course. In the present report, we describe a slowly progressive form of this disease associated with this mutation. We also carried out linkage analysis studies on four unrelated families, including the two previously reported families (Dicapua and Patwa 2014), to confirm this is a founder mutation in Hispanic LGMD2C patients from Puerto Rico.

All four patients in this report had onset of disease in the first decade of life. The pattern of proximal weakness, calf hypertrophy, and scapular winging observed in our cohort are features that are commonly seen in LGMD2C patients. The progression of weakness observed in two of our patients, Patients 1 and 2, is slower than the North African and Gypsy cases of LGMD2C resulting from founder mutations (Ben Hamida et al. 1983; Piccolo et al. 1996; Lasa et al. 1998; Kefi et al. 2003). If we were to apply the severity score described by Kefi et al. (2003) to our patients, they would fall in the mild form of disease that corresponds to the loss of less than one functional grade in the lower extremities per 2 years of disease duration. This relatively slowly progressive course of disease was also observed in a large consanguineous Dutch family (705T>C, p.Leu193Ser), in which five subjects remained ambulatory into their fourth decade of life (van Kooi et al. 1998). Although, *γ*-sarcoglycan is highly expressed in the myocardium (Noguchi et al. 1995), cardiac involvement in patients with LGMD2C is quite variable. Earlier descriptions of global cardiac dysfunction (Ben Hamida et al. 1983) were followed by reports of subclinical myocardial changes(Calvo et al. 2000) and others of normal cardiac function (Melacini et al. 1999). None of our patients had electrographic changes on EKG at the time of diagnosis and cardiac function by echocardiogram was normal.

Complete absence of staining to the *γ*-sarcoglycan antibody in our cohort is not surprising as it concurs with previous reports of genetically confirmed LGMD2C, where selective absence or greater reduction in *γ* -sarcoglycan in muscle biopsy is generally predictive of a *SGCG* mutation (McNally et al. 1996a,b; Moore et al. 2006; Klinge et al. 2008). However, partial deficiencies of *γ* -sarcoglycan with preservation of the other sarcoglycan proteins can occur (Crosbie et al. 1999; Bonnemann et al. 2002).

The subjects in this report are homozygous for a missense mutation in exon 8 of the *SGCG* gene. The amino acid change from glutamic acid to lysine (E263K) that results from this mutation occurs in the extracellular carboxyl terminus of *γ*-sarcoglycan. Microdeletions in this domain of the *SGCG* gene have been associated with complete loss of *γ*-sarcoglycan immunostaining in the muscle and partial reduction in other sarcoglycans (McNally et al. 1996a). Given this region contains conserved cysteine residues, it is hypothesized that disruption of the carboxyl terminus of the *γ*-sarcoglycan protein results in destabilization of the sarcoglycan complex (McNally et al. 1996a).

Although our patients identified themselves as non-white Hispanics from the island of Puerto Rico, this does not define the genetic ancestry of this founder mutation. The current population of Puerto Rico is an amalgam of three ancestral populations: Taino Indians, Europeans, and Africans. Haplogrouping of 800 random samples representative of the population of Puerto Rico, showed mtDNA lineages were 61.3% Amerindian, 27.2% African, and 11.5% as European (Martinez-Cruzado et al. 2005). Also, analysis of Taino Indian remains, has shown very low genetic diversity, inferring a founder effect that predates European contact with this island (Lalueza-Fox and Calderon 2001). This finding, along with the additional observations of ancestry-based assortative mating among Puerto Ricans (Risch et al. 2009), suggests that the E263K mutation likely originates from the Taino Indian heritage rather than the Spanish/European.

To our knowledge this is the first report to provide unequivocal evidence that the missense mutation c.787G>A in exon 8 of the *SGCG* gene is a founder mutation in Puerto Rican Hispanics by linkage analysis. A recent case report of two Puerto Rican siblings with this mutation and early loss of ambulation strengthens our findings of a founder effect in this population and variability in the LGMD2C phenotype (Dicapua and Patwa 2014) (Table2). Given the restricted population of Puerto Rico, this founder effect may influence disease prevalence in that region.

**Table 2:** Clinical phenotype of reported Puerto Rican Hispanics with LGMD2C

Patient	Age (years)	Disease onset	Ambulation	Calf hypertrophy	Contractures	Cardiac	Level of *γ*-sarcoglycan
Pt 1	11	6 years	Yes	Yes	No	EF >55%	Absent
Pt 2	18	9 years	Yes	Yes	No	EF >55%	Absent
Pt 3	18	7 years	No (lost at 10 years)	Yes	Yes	EF 50–55%	Absent
Pt 4	4	2 years	Yes (at 4 years)	Yes	No	n/a	Absent

n/a, data not available.
